# *LAPTM5*–CD40 Crosstalk in Glioblastoma Invasion and Temozolomide Resistance

**DOI:** 10.3389/fonc.2020.00747

**Published:** 2020-06-05

**Authors:** Anne Berberich, Frederik Bartels, Zili Tang, Maximilian Knoll, Sonja Pusch, Nanina Hucke, Tobias Kessler, Zhen Dong, Benedikt Wiestler, Frank Winkler, Michael Platten, Wolfgang Wick, Amir Abdollahi, Dieter Lemke

**Affiliations:** ^1^German Cancer Research Center (DKFZ), German Cancer Consortium (DKTK), Heidelberg, Germany; ^2^Department of Neurology, University of Heidelberg Medical School and National Center for Tumor Diseases (NCT), Heidelberg, Germany; ^3^Division of Molecular and Translational Radiation Oncology, Heidelberg Ion Therapy Center (HIT), German Cancer Research Center (DKFZ), Heidelberg Institute of Radiation Oncology (HIRO), University of Heidelberg Medical School and National Center for Tumor Diseases (NCT), Heidelberg, Germany; ^4^Department of Neurosurgery, Tongji Medical College, Tongji Hospital, Huazhong University of Science and Technology, Wuhan, China; ^5^Department of Neuroradiology, Klinikum rechts der Isar der Technischen Universität, Munich, Germany; ^6^Department of Neurology, Medical Faculty Mannheim, Heidelberg University, Heidelberg, Germany; ^7^DKTK Clinical Cooperation Unit Neuroimmunology and Brain Tumor Immunology, German Cancer Research Center (DKFZ), Heidelberg, Germany

**Keywords:** *LAPTM5*, CD40, NFκB, temozolomide, glioblastoma

## Abstract

**Background:** Glioma therapy is challenged by the diffuse and invasive growth of glioma. Lysosomal protein transmembrane 5 (*LAPTM5*) was identified as an invasion inhibitor by an *in vivo* screen for invasion-associated genes. The aim of this study was to decipher the function of *LAPTM5* in glioblastoma and its interaction with the CD40 receptor which is intensively evaluated as a target in the therapy of diverse cancers including glioma.

**Methods:** Knockdown of *LAPTM5* was performed in different glioma cell lines to analyze the impact on clonogenicity, invasiveness, sensitivity to temozolomide chemotherapy, and tumorigenicity *in vitro* and *in vivo*. An expression array was used to elucidate the underlying pathways. CD40 knockdown and overexpression were induced to investigate a potential crosstalk of *LAPTM5* and CD40. *LAPTM5* and CD40 were correlated with the clinical outcome of glioma patients.

**Results:** Knockdown of *LAPTM5* unleashed CD40-mediated NFκB activation, resulting in enhanced invasiveness, clonogenicity, and temozolomide resistance that was overcome by NFκB inhibition. *LAPTM5* expression correlated with better overall survival in glioblastoma patients depending on CD40 expression status.

**Conclusion:** We conclude that *LAPTM5* conveyed tumor suppression and temozolomide sensitation in CD40-positive glioblastoma through the inhibition of CD40-mediated NFκB activation. Hence, *LAPTM5* may provide a potential biomarker for sensitivity to temozolomide in CD40-positive glioblastoma.

## Introduction

Despite relevant improvements in the molecular understanding of glioma, treatment options remain limited for glioma patients. Glioma cells infiltrate diffusely throughout the whole brain, forming a functional tumor network that contribute to therapy resistance and recurrence after standard multimodal therapy including surgery, irradiation, and chemotherapy with temozolomide (1, 2). The complex processes underlying tumor cell migration and invasion have been studied intensively, but effective treatments targeting the pro-invasive mechanisms are still required (3–6).

Screening for invasion-associated genes identified lysosomal protein transmembrane 5 (*LAPTM5*) as a relevant gene involved in the invasion of glioblastoma. *LAPTM5*, which is localized in the lysosomal membrane (7), was downregulated in a variety of human cancer cells, suggesting that the inactivation of *LAPTM5* plays a role in tumorigenesis (8). Low *LAPTM5* expression was significantly correlated with poor prognosis in patients with esophageal squamous cell carcinoma and non-small cell lung cancer (NSCLC) (8). Furthermore, *LAPTM5*-mediated programmed cell death was involved in the spontaneous regression of neuroblastoma cells (9). In HeLA cells, the ectopic overexpression of *LAPTM5* induced apoptosis *via LAPTM5*-mediated lysosomal pathways (10). In addition, *LAPTM5* was rapidly and transiently repressed by treatment with CD40 ligand in B-cells (11), which resulted in the alternative name CD40-ligand activated transcript. Recently, CD40 was discovered as an emerging target in cancer immunotherapy, and CD40 agonistic therapies are being evaluated in antitumor trials for different cancer types including non-Hodgkin's lymphoma, advanced urothelial carcinoma, malignant melanoma, pancreatic adenocarcinoma, renal cells carcinoma, NSCLC, and glioma (12). The interaction of CD40 and the CD40 ligand (CD40L or CD154) has been shown to exert a plethora of immune-stimulating effects in the innate and the adaptive immune system (Supplemental Figure 1) (13–19). In addition, substantial CD40 expression was detected in a variety of common solid cancer types including bladder cancer (102/131, 78%), melanoma (41/71, 57.7%), breast cancer (53%), lung cancer (67/129, 51.9%), colon cancer (87/110, 79%) as well as B-lineage malignancies (14–19). CD40 expression was frequently shown to be associated with prolonged survival; however, contrary results were evident in lung and esophageal cancer where CD40 expression correlated with poor prognosis (17–20). In glioma, only scarce and partially contradictory data exist about *LAPTM5* and CD40 (20, 21). Therefore, the aims of this study were to characterize the role of *LAPTM5* in glioblastoma, to elucidate its interaction with CD40, and to analyze the underlying signaling pathways involved in tumorigenicity.

## Materials and Methods

### Glioblastoma Cells and Culture Conditions

All glioblastoma cell lines [LN-229 (RRID:CVCL_0393), LN-308 (RRID:CVCL_0394), U-87MG ATCC (RRID:CVCL_0022), LN-428 (RRID:CVCL_3959), and T98G (RRID:CVCL_0556)] were purchased from the American Type Culture Collection (Manassas, VA, USA) and cultured under standard conditions. Glioblastoma-initiating cells (T325, T269, and S24) were established from freshly dissected glioblastoma tissue from adult patients after informed consent and cultured in neurosphere medium (DMEM/F12 medium, Life Technologies, Carlsbad, CA, USA) supplemented with B27 supplement, heparin (5 μg/ml), basic fibroblast growth factor (20 ng/ml), and epidermal growth factor (20 ng/ml) (22). All cell lines tested have an isocitrate dehydrogenase wild-type status. The SNP profiles matched known profiles or were unique. The cell lines were authenticated using Multiplex Cell Authentication by Multiplexion (Heidelberg, Germany) as previously described (23). Absence of mycoplasma infection is screened for on a regular basis.

### Knockdown of Gene Expression

The knockdown of *LAPTM5* gene expression was induced using a commercial set of five lentiviral small hairpin RNAs (shRNA) targeting the 3′UTR region of the *LAPTM5* transcript. The most effective construct was used for the experiments (Mission®, shRNA TRCN0000123149, Clone ID: NM_006762.1-1614s1c1) (Sigma Aldrich, Taufkirchen, Germany). The non-targeting shRNA lentiviral construct (Mission® SHC002, Sigma Aldrich) was used as a control (24). Furthermore, to reevaluate the experiments with another shRNA system, the knockdown of *LAPTM5* was also performed with pZIP-SFFV-RFP (shERWOOD-UltramiR shRNA Target Gene Set, cat. no.: TLHSU1400-7805-pZIP-SFFV-RFP-GVO-TRI, Transomic Technologies, Huntsville, AL 35806, USA). The knockdown of CD40 gene expression was induced using a commercial green fluorescent protein (GFP)-tagged lentiviral shRNA interfering system (Mission shRNA TRCN000038715, ID: NM_011611). After transduction, the cells were selected by treatment with neomycin and fluorescence-activated cell sorting (FACS) of GFP-positive cells. The non-targeting GFP-shRNA lentiviral construct (Mission® SHC004, Sigma Aldrich) was used as a control.

The transient knockdown of CD40 was performed with siRNA transfection (Sigma Aldrich) using lipofectamine (Invitrogen, Carlsbad, CA, USA) according to the manufacturer's protocol. All knockdown experiments were controlled with the respective non-targeting shRNA lentiviral construct. In double-knockdown experiments, two respective non-targeting shRNA lentiviral constructs were used as control. All different control knockdown constructs were named *v-control* (vector control).

### CD40 Overexpression

CD40-overexpressing cells were generated after the transfection of cells with pcDNA3 control or pcDNA3 CD40 plasmid, as generously provided by M. Weller (Zurich, Switzerland). The CD40-vector was generated by the amplification of cDNA prepared from EJ cells using CD40-specific primers (forward primer: 5′-CTGGTCTAAGCTTGCCATGGTTC-3′ and reverse primer: 5′-TGGGTGGCGGCCGCCTCACT-3′) which were inserted into the HindIII/NotI sites of pcDNA3 (25). After transfection, the CD40-positive cells were sorted by flow cytometry. In cells with combined CD40 overexpression and *LAPTM5* knockdown, the control cells were transfected with empty pcDNA3 and transduced with the respective non-targeting shRNA lentiviral construct. All control constructs were named v-control (vector control).

### Quantitative Real-Time PCR

RNA extraction, cDNA synthesis, and quantitative real-time PCR (qRT-PCR) were performed as previously described (26). All results were normalized to glyceraldehyde-3-phosphate dehydrogenase serving as a housekeeping gene (27). The primer sequences are listed in Supplemental Table 1.

### Whole-Exome mRNA Analysis

Gene expression analysis was performed to compare the *LAPTM5* knockdown in U87MG cells with the respective controls (28). From each cell line, three independent total RNA samples were used for the microarray. The sample analysis was performed on an illumina HT 12 microarray chip at the genomics core facility (German Cancer Research Center, Heidelberg, Germany). The differentially expressed genes were identified after adjustment for multiple testing using the Benjamini–Hochberg correction and a significance threshold of *p* < 0.05. Ingenuity Pathway Analysis (Ingenuity Systems, Redwood City, CA, USA) was used for protein network and pathway analysis. The experiment ArrayExpress accession code is E-MTAB-6316. For the gene set enrichment analysis (GSEA), the GSEA software tool was downloaded from the homepage of the Broad Institute (http://software.broadinstitute.org/gsea). The hallmark gene sets from the Molecular Signature Database v6.1 were used for the exploratory testing of pathway enrichments in the full dataset of genes in the microarray. The number of gene set permutations was set to 1,000.

### Matrigel Invasion Assay

Glioblastoma cell invasion was measured *in vitro* with the Boyden chamber assay (BD Biosciences, San Jose, CA, USA) (29). The migrated cells were stained with 4,6-diamidino-2-phenylindol (Vector Laboratories, Burlingame, CA, USA), scanned with Cell Observer (Zeiss, Oberkochen, Germany), and counted using ImageJ software (Bethesda, MD, USA). The number of migrated cells was corrected by proliferation control, which was assessed in parallel by crystal violet staining.

### Limiting Dilution Assay

The clonogenic potential of glioblastoma cells was evaluated with the Limiting Dilution Assay as previously described (22, 30). Four different dilutions (300, 50, eight, and one cell per well) were seeded in a 96-well plate and cultured for 28 days (24 wells per dilution). The number of wells with colony formation was counted. The cells were treated with 2.5 μg/ml JSH-23 and 10 μM temozolomide (U87MG) or 5 μM temozolomide (LN229 and LN308) depending on the intrinsic sensitivity to the temozolomide treatment of the respective cell lines.

### Immunoblot Analysis

Whole cell lysates were prepared as described previously (26). The antibodies used are listed in Supplemental Table 2. Equal protein loading was controlled with mouse α-tubulin (1:5,000, Sigma Aldrich, USA) staining.

### Flow Cytometry Analysis

For flow cytometry, the cells were dissociated with accutase, washed, stained with CD40-FITC or the respective isotype control, and analyzed with a BD-FACS Canto II flow cytometer (BD Biosciences, USA); final data were processed with the FlowJo flow cytometry analysis software (Tree Star Inc., USA). Specific fluorescence intensity was calculated by using the mean fluorescence signal of CD40 divided by the mean fluorescence isotype signal.

### Xenograft Mouse Model

All works involving animals were approved by the governmental authorities (Aktenzeichen: 35-9185.81 G-136/12 Regierungspräsidium Karlsruhe, Germany) and performed in accordance with the German animal protection law. To test the tumorigenicity *in vivo*, a 200-μl suspension of 1 × 10^7^ tumor cells in 100 μl phosphate-buffered saline and 100 μl Matrigel (BD Biosciences, San Jose, CA, USA) was injected s.c. in the flank of 8-week-old female CD1 nu/nu mice (Charles River Laboratories, Sulzfeld, Germany) (31). The tumor volume was calculated by (length × width × width)/2. The mice were sacrificed in accordance with the German animal protection law when they showed symptoms of disease or when the maximal tumor diameter reached 1.5 cm. Treatment (oral gavage) with 21 mg/kg temozolomide in methylcellulose *vs*. methylcellulose alone in controls started on day 14 after tumor implantation and was given for 7 days every 24 h.

### Methylation and mRNA Expression Correlation Analysis

The CpG sites in proximity to the *LAPTM5* gene location were identified using the UCSC Genome Browser available at https://genome.ucsc.edu/. The dataset including 27k methylation data and mRNA expression data was downloaded from The Cancer Genome Atlas (TCGA), available at http://cancergenome.nih.gov/. The Pearson correlation coefficient of methylation level at the indicated CpG site and *LAPTM5* mRNA expression was calculated using the software R.

### 5-Azacytidin Demethylation

Human glioma-initiating cell lines T325, T269, and T1 were incubated with 5 μM 5-azacytidin (Sigma-Aldrich, St. Louis, MO, USA) dissolved in dimethyl sulfoxide (DMSO) for 5 days with a complete change of the growth medium and with fresh 5-azacytidin added every day. The respective control cells were incubated with DMSO only. The cells were then harvested and *LAPTM5* mRNA expression was measured using qRT-PCR.

### Clinical Survival Analysis

To assess the prognostic value of *LAPTM5* in high CD40-expressing tumors, methylation array (450 k) and RNASeq data from the TCGA-Glioblastoma Multiforme (GBM) project were used. For the selection of a reasonable cutoff for CD40 expression, similarity of patients was derived from methylation-based correlation (cluster boostrap of the correlation matrix, pvclust package) using CpG probes significantly associated with CD40 gene expression (linear model, Bonferroni adjusted *p* < 0.05, *n* = 24), resulting in two main clusters. Using a receiver operating characteristic (ROC) analysis, a cutoff between both clusters was selected based on maximization of the Youden index. The respective cutoff was then applied to the RNASeq cohort to select CD40 high-expressing tumors. Survival analysis was performed with parametric survival regressions, assuming a Weibull distribution.

### CIBERSORT Analysis

CIBERSORT allows the estimation of immune cell fractions from bulk expression data (32). Preprocessed RNASeq data was used to calculate immune cell fractions.

### Statistical Analysis

All *in vitro* experiments reported represent at least three replicated independent experiments containing triplicates or more. A statistical analysis of differences between mean values was performed using Student's *t*-test. The differences between estimated clonogenic cell frequencies analyzed by the ELDA web tool were compared using output of tests for pair-wise differences in active cell frequency between groups (33). A two-way ANOVA model was used for interaction analysis between two factors, e.g., gene knockdown and inhibiting agent. If not stated otherwise, all statistical analyses were performed using GraphPad Prism version 6.00 for MacOS X, GraphPad Software, La Jolla, CA, USA. All *p*-values were two-tailed. A *p* < 0.05 was considered significant, with asterisks marking the different levels of significance: ns, *p* ≥ 0.05; ^*^p < 0.05; ^**^p < 0.01. TCGA data analyses were performed in R (v3.6.1) (34). Survival analyses and ROC analyses were performed using the survival (35) and dataAnalysisMisc packages (36).

## Results

### *LAPTM5* Inhibits Tumorigenicity

An *in vivo* screen for invasion-associated genes in glioblastoma (**Figure 6A**, further unpublished data) identified *LAPTM5* as a highly anti-invasive gene in glioblastoma. Briefly, 47,400 transcripts by ~200,000 shRNA sequences were transduced in U87 MG cells to identify new invasion-associated genes. Here *LAPTM5* was found to be downregulated in the invasive front of U87MG-derived orthotopically implanted tumors.

**Figure 1:**
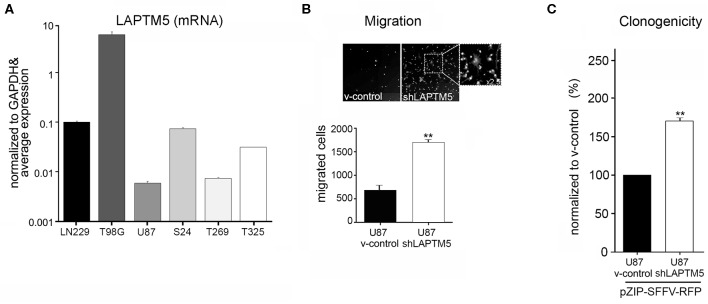
*LAPTM5* acts as tumor suppressor gene in glioma. **(A)** *LAPTM5* mRNA expression in glioblastoma cell lines (U87MG, LN229; T98G) and in primary glioma-initiating cells S24, T269, and T325 analyzed by quantitative real-time PCR and presented in relation to the average *LAPTM5* expression in the tested cells. **(B)** *LAPTM5* inhibits the invasion of U87MG cells. Representative image fields (×10 magnification) of migrated cells in Boyden Chamber invasion assay (upper figure) as well as total number of migrated cells (lower figure) of three independent experiments demonstrate a significantly reduced migration of *LAPTM5* expressing (v-control) compared to *LAPTM5* knockdown cells. **(C)** *LAPTM5* inhibits the clonogenicity of U87MG cells. *LAPTM5*-knockdown U87MG cells show a significantly higher clonogenicity than the respective *LAPTM5* expressing (v-control) cells. Figures represent the mean value of three independent experiments. *p < 0.05, **p < 0.01.

*LAPTM5* was relevantly expressed in all glioblastoma cell lines and primary glioblastoma cells tested (Figure 1A). Methylation *in silico* analysis further revealed that *LAPTM5* expression was negatively regulated by methylation of the CpG sites Cg10001720 and Cg12732155, which was substantiated by treatment with the demethylating agent 5-aza-2′-deoxycytidine. This treatment induced > 6-fold upregulation of *LAPTM5* mRNA expression in primary glioblastoma cell lines (Supplemental Figure 2).

Silencing of *LAPTM5* (Supplemental Figure 3A) confirmed its anti-invasive function in U87MG cells *in vitro*. The *LAPTM5* knockdown (sh*LAPTM5*) cells demonstrated significantly increased invasiveness by a factor of ~3 compared to the respective control cells (v-control) (Figure 1B). Additionally, *LAPTM5* inhibited the clonogenicity of U87MG glioblastoma cells as *LAPTM5* knockdown cells demonstrated a significantly higher clonogenic capacity compared to the respective vector control cells (Figure 1C). This pro-clonogenic phenotype of *LAPTM5* knockdown cells was confirmed with two different knockdown constructs (Supplemental Figure 3B).

### *LAPTM5* Function Is CD40 Dependent

**Figure 2:**
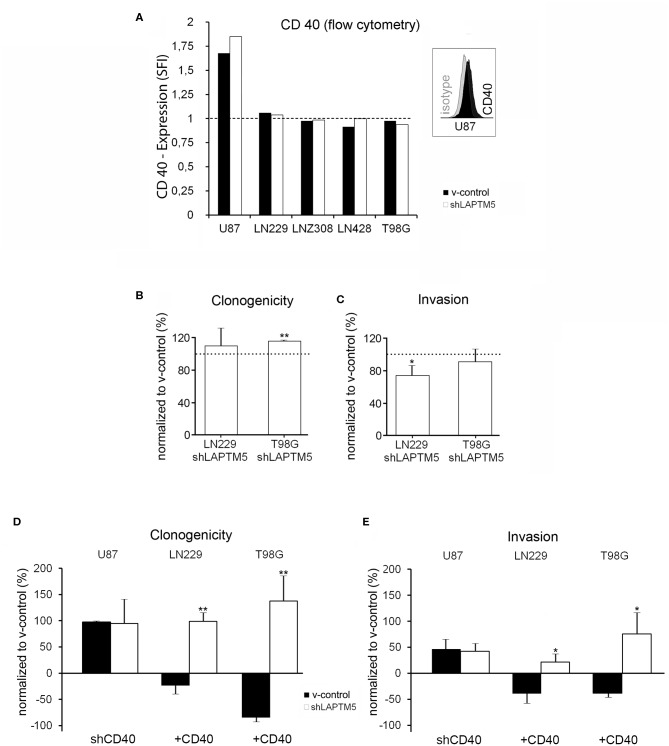
Tumor-suppressive functions of *LAPTM5* were dependent on CD40 expression. **(A)** Flow cytometric analysis after CD40 surface staining illustrates that only U87MG v-control and sh*LAPTM5* cells are positive for CD40, whereas the other glioblastoma cell lines were negative for CD40 membrane expression and did not show a specific fluorescence index clearly >1 when dividing the mean fluorescence of the CD40 staining by the mean fluorescence of the respective isotype control. **(B)** Clonogenicity in the CD40-negative LN229 cells did not relevantly differ between v-control control and sh*LAPTM5* cells and was slightly higher in the CD40-negative T98G cells silenced for *LAPTM5* compared to the respective v-control control cells. The graphs show the clonogenicity of sh*LAPTM5* normalized to the respective v-control control cells. **(C)** Knockdown of *LAPTM5* led to a slightly reduced invasiveness of LN229 cells and did not relevantly alter the invasiveness in T98G cells compared to the respective *LAPTM5*-expressing (v-control) cells. The graphs visualize the invasiveness of sh*LAPTM5* cells normalized to the respective v-control control cells. **(D,E)** Graphs show the clonogenicity **(D)** and invasiveness **(E)** depending on CD40 expression status in relation to the respective v-control control cells. The dotted lines signify no change in relation to v-control control cells. The figures represent the mean value of three independent experiments. *p < 0.05, **p < 0.01. In CD40-positive U87MG cells, silencing of CD40 results in an equalized clonogenicity and invasiveness of v-control and *LAPTM5* knockdown cells and thereby mirrors the phenotypes of the CD40-negative LN229 and T98G v-control and sh*LAPTM5* cells **(B,C)**. Exogenous overexpression of CD40 in the CD40-negative LN229 and T98G cells results in a significant higher clonogenicity and invasiveness of *LAPTM5* knockdown in relation to the respective v-control control cells and thereby confirms the results observed in the CD40-positive U87MG cells (demonstrated in Figures 1B,C).

*LAPTM5* was previously shown to be regulated by the CD40 receptor in immune cells and CD40 is highly expressed in up to 40% of glioblastoma (11). In order to identify the underlying molecular mechanisms of *LAPTM5* function, we aimed to explore the CD40–*LAPTM5* interaction in glioblastoma. For that, established glioblastoma cell lines were examined for membrane CD40 expression by flow cytometry, revealing only U87MG cells to be clearly positive for CD40 expression (Figure 2A). To test whether this is an artifact due to medium-related conditions or a problem of established cell lines in general, the glioma-initiating cells S24, T1, T269, T325, ZH305, ZH161, WJ, KNG002, MM, and PJ (22) were also tested for CD40 expression by flow cytometry but were all negative (data not shown).

Interestingly, in contrast to CD40-expressing U87MG cells, *LAPTM5* hardly influenced the clonogenicity and the invasiveness in the CD40-negative LN229 and T98G cells. Clonogenicity was not enhanced in *LAPTM5*-depleted LN229 cells and only by ~15% in *LAPTM5-*knockdown T98G cells (Figure 2B). Invasiveness even had a tendency to be reduced in *LAPTM5*-knockdown LN229 and T98G cells (Figure 2C), suggesting that CD40 expression might be required for *LAPTM5*-mediated effects. Indeed the knockdown of CD40 in U87MG v-control and sh*LAPTM5* cells (Supplemental Figure 4A) resulted in enhanced clonogenicity by ~100% and invasiveness by ~50% in U87MG, but the difference between the control and the sh*LAPTM5* cells vanished (Figures 2D,E, left row) and thereby mirrored the situation of the CD40-negative LN229 and T98G cells (Figures 2B,C).

To further substantiate the CD40 dependency of *LAPTM5*-mediated anti-tumorigenic effects, CD40 was overexpressed in the CD40-negative LN229 and T98G v-control and sh*LAPTM5* cells (Supplemental Figures 4B,C). Indeed CD40 overexpression resulted in higher clonogenicity and enhanced invasiveness of sh*LAPTM5* LN229 (clonogenicity + ~100%; invasiveness + ~25%) and T98G cells (clonogenicity + ~140%; invasiveness + ~75%) compared to the respective v-control vector control cells which became less clonogenic and invasive (Figures 2D,E). These results were consistent with the effects observed in U87MG cells. In summary, *LAPTM5*-mediated anti-tumorigenic effects were dependent on CD40-expression: CD40 knockdown blocked and CD40 overexpression unleashed *LAPTM5*-mediated anti-tumorigenic effects in three different cell lines.

### *LAPTM5* Sensitizes to Temozolomide in CD40-Expressing Glioblastoma Cells

**Figure 3:**
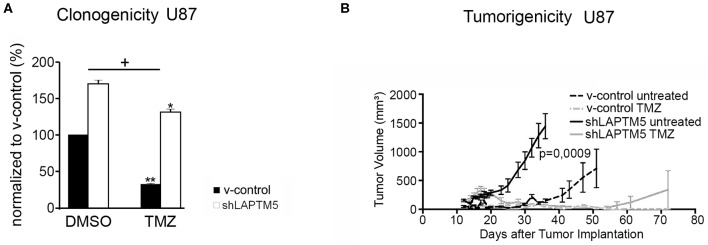
*LAPTM5*-sensitized CD40-positive U87MG cells to temozolomide chemotherapy. **(A)** *LAPTM5* sensitizes U87MG cells to temozolomide treatment, whereas *LAPTM5* knockdown cells were significantly more resistant against temozolomide chemotherapy. Figures elucidate the differences in clonogenicity in relation to v-control control cells treated with DMSO control. The asterisk shows a significant effect of the treatment on the cell line (*p < 0.05, **p < 0.01); the plus sign points out that the sh*LAPTM5* cells are more resistant to temozolomide compared with control cells as the difference in clonogenicity is more prominent after treatment as measured by ANOVA. **(B)** *LAPTM5* relevantly delays the tumor growth of U87MG glioblastoma cells *in vivo* and sensitizes tumors to temozolomide treatment. The figure represents the changes in mean tumor volume of five mice per group over an observation time of 100 days.

To examine the relevance of *LAPTM5* in the mediation of treatment resistance to standard therapy for glioblastoma patients, the efficacy of temozolomide chemotherapy was further analyzed according to *LAPTM5* status in CD40-expressing U87MG cells. The *LAPTM5*-expressing U87MG cells were more sensitive to temozolomide treatment compared to the *LAPTM5* knockdown cells. Temozolomide reduced clonogenicity by 68% in v-control control cells but only by 23% in sh*LAPTM5* U87MG cells (Figure 3A).

These *LAPTM5*-mediated effects were further confirmed *in vivo. LAPTM5* inhibited the tumorigenicity of implanted U87MG cells. The tumor volumes of sh*LAPTM5* tumors already exceeded the size of 500 mm3 after 27 days, which was taken as an undoubted evidence of tumor growth, whereas tumors with *LAPTM5* expression did not reach this tumor size earlier than 56 days after tumor cell implantation. Furthermore, temozolomide treatment relevantly and sustainably inhibited tumor growth in *LAPTM5*-expressing tumors without any evidence for tumor recurrence within the observation time of 100 days. In contrast, tumor recurrence was observed in one out of five sh*LAPTM5* tumors after initial shrinkage due to temozolomide treatment (Figure 3B), supporting that *LAPTM5* sensitized the CD40-positive U87MG tumor cells to temozolomide.

### *LAPTM5* Conveys Tumor-Suppressive Effects *via* Inhibition of CD40-Mediated NFκB Signaling

**Figure 4:**
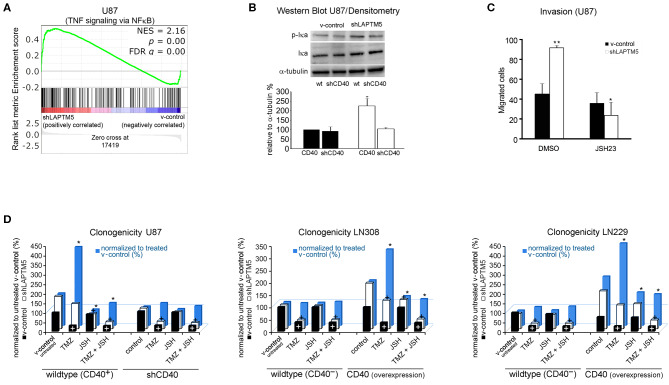
*LAPTM5* conveys tumor-suppressive effects by inhibition of NFκB-pathway *via* CD40. **(A)** Gene set enrichment analysis revealed a significantly higher activation of TNF-α signaling *via* the NFκB pathway in *LAPTM5* knockdown compared to v-control U87MG cells. **(B)** Immunoblot analysis confirmed the activation of NFκB pathway in sh*LAPTM5* cells as demonstrated by the higher phosphorylation of p-Iκa in *LAPTM5* knockdown compared to the respective *LAPTM5*-expressing (v-control) U87MG cells. NFκB pathway activation could not be substantiated after additional knockdown of CD40 in sh*LAPTM5* and v-control U87MG cells. The figure demonstrates one representative immunoblot analysis (upper panel) and the quantification of immunoblot analysis performed with image J of three independent experiments (lower panel). The asterisk signifies that sh*LAPTM5* wild type has a significantly higher p-Iκa-level than v-control wild-type cells (*p < 0.05). **(C)** The functional relevance of NFκB activation in *LAPTM5* knockdown cells was further substantiated by treatment with the NFκB inhibitor JSH-23, which specifically reduced the increased invasiveness of *LAPTM5* knockdown cells, whereas it did not relevantly alter the invasiveness in v-control cells (*p < 0.05, ** *p* < 0.01). **(D)** The graphs demonstrate the effects of temozolomide treatment, NFκB inhibition with JSH-23, and the combination of both treatments depending on the CD40 expression status of the respective cell lines. For that, CD40 was silenced in the CD40-positive U87MG cells or exogenously overexpressed in the CD40-negative LN229 and LN308 cell lines, and clonogenicity was analyzed after the respective treatments. The clonogenicity of v-control (white columns) and sh*LAPTM5* (black columns) cells are demonstrated in relation to the v-control wild-type cells treated with DMSO control. The plus sign signifies that there was a significant therapy effect measured by ANOVA compared with DMSO (untreated) control (at least *p* < 0.05). The blue bars show the ratio of sh*LAPTM5* relative to v-control cells for every single treatment (black in relation to white column for each treatment) to compare and illustrate the efficacy of each therapy in sh*LAPTM5* and control cells; the asterisk points out that the sh*LAPTM5* cells react differently from v-control cells when compared to the ratio sh*LAPTM5*/v-control in DMSO control treatment as measured by ANOVA (at least *p* < 0.05). The *LAPTM5* knockdown led to a higher clonogenicity in CD40-positive glioblastoma cells and to a higher resistance against temozolomide treatment. The *LAPTM5* knockdown cells were particularly sensitive to NFκB inhibition by JSH-23 in CD40-positive glioblastoma cells. In addition, NFκB inhibition re-sensitized the resistant *LAPTM5* knockdown cells to treatment with temozolomide. In the CD40-negative glioblastoma cells, LN229 and LN308 as well as U87MG cells silenced for CD40; no differences were observed between the respective v-control and *LAPTM5* knockdown cells, and JSH-23 had no effect on clonogenicity.

To understand the molecular mechanisms by which *LAPTM5* inhibited invasion and tumorigenicity and sensitized to temozolomide treatment, a gene set enrichment analysis of whole-exome expression profiled U87MG cells was performed and compared with U87MG cells silenced for *LAPTM5* expression. Here gene network and pathway analysis showed a strong upregulation of TNF-α signaling *via* the NFκB pathway in sh*LAPTM5* cells (Figure 4A; Supplemental Table 3), suggesting an inhibitory effect of *LAPTM5* on NFκB signaling. An immunoblot analysis confirmed the activation of the NFκB pathway in sh*LAPTM5* U87MG cells, demonstrated by an increased expression of phosphorylated IκBα (Figure 4B). Of note is that CD40 expression was mandatory in inducing NFκB signaling in sh*LAPTM5* U87MG cells as pathway activation was no longer observed after the additional knockdown of CD40 in these cells (Figure 4B). On the functional level, JSH-23, a specific inhibitor of the NFκB pathway, particularly reduced the cell invasiveness in sh*LAPTM5* but did not alter the invasiveness of v-control U87MG cells, indicating that *LAPTM5* knockdown promotes higher cell invasiveness *via* NFκB pathway activation (Figure 4C).

Similarly, only U87MG *LAPTM5* knockdown cells were highly sensitive to treatment with the NFκB inhibitor JSH-23, resulting in a significant inhibition of clonogenicity, which was not demonstrated in control cells (Figure 4D, left panel).

As *LAPTM5* was shown to sensitize CD40-positive U87MG glioblastoma cells to temozolomide and inhibited the NFκB pathway, the functional role of potential NFκB-mediated temozolomide resistance in *LAPTM5* knockdown cells as well as the connection with CD40 expression was further investigated.

Consistent with the previous data (Figure 3A), temozolomide relevantly reduced clonogenicity in v-control U87MG cells, whereas sh*LAPTM5* cells were more resistant against temozolomide treatment. Moreover, NFκB inhibition specifically sensitized sh*LAPTM5* U87MG cells to temozolomide treatment, resulting in a comparable inhibitory efficacy of temozolomide on clonogenicity in *LAPTM5* knockdown and v-control control cells (Figure 4D, left panel).

Importantly, the differences in response to temozolomide and JSH-23 treatment vanished after knockdown of CD40 in U87MG v-control and sh*LAPTM5* cells. After CD40 knockdown, not only the higher clonogenicity but also the temozolomide resistance effect of the *LAPTM5* knockdown cells could not be observed anymore. Furthermore, no relevant response to the inhibition of the NFκB pathway with JSH-23 treatment was seen in *LAPTM5* and CD40 double-knockdown cells (Figure 4D, left panel).

Aiming to exclude potential cell line artifacts, CD40-negative LN229 as well as LN308 control and sh*LAPTM5* cells were further examined with respect to responsiveness to temozolomide and NFκB inhibition. Consistent with the data in CD40-silenced U87MG cells, the knockdown of *LAPTM5* did not confer resistance against temozolomide. Moreover, NFκB inhibition had no relevant effect on sh*LAPTM5* or v-control control cells regardless of co-treatment with temozolomide. However, the overexpression of CD40 induced a marked resistance against temozolomide in LN229 and LN308 sh*LAPTM5* cells, but not in v-control control cells. Of note is that again only CD40-positive sh*LAPTM5* cells showed relevant sensitivity to NFκB inhibition, which was able to level the sh*LAPTM5*-induced temozolomide resistance (Figure 4D, middle and right panel). In conclusion, these data demonstrate that *LAPTM5* mediates the sensitivity to temozolomide by the inhibition of CD40-induced NFκB pathway activation in CD40-positive glioblastoma cells.

### The Prognostic Value of *LAPTM5* Is Dependent on CD40 Abundance

**Figure 5:**
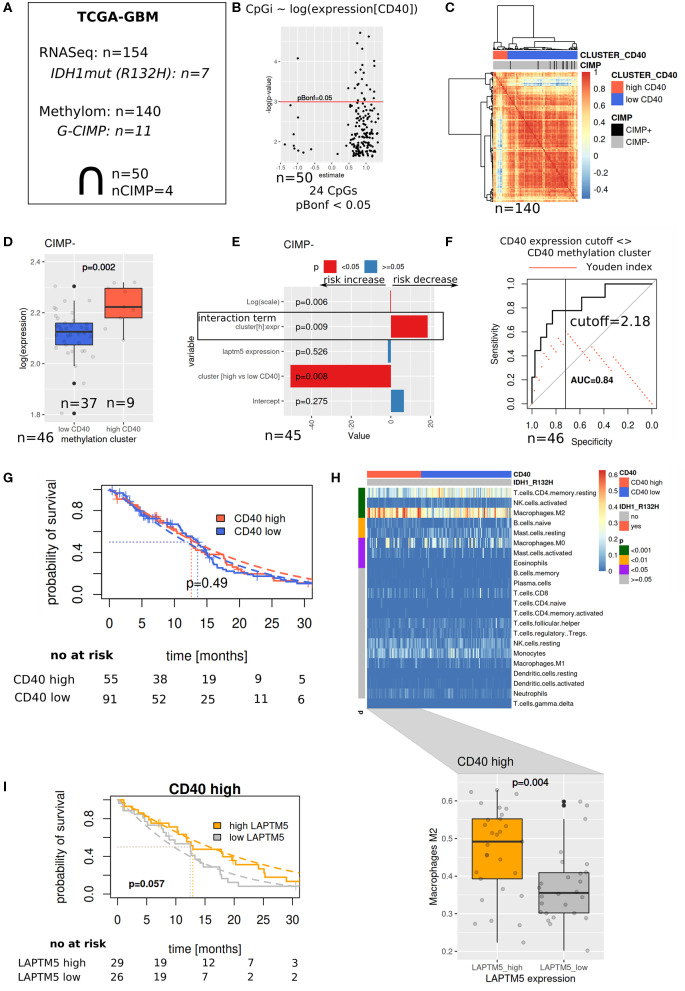
The prognostic value of *LAPTM5* is dependent on CD40 abundance. **(A)** Evaluated The Cancer Genome Atlas (TCGA)-glioblastoma multiforme methylome (450 k array) and RNASeq datasets. **(B)** Associations between CD40 expression and methylation array probes (linear model) with Bonferroni adjusted *p* < 0.05. **(C)** Patient similarity (Pearson correlation matrix) based on CpGs selected in **(B)** and used for CD40 cutoff identification in the complete TCGA methylome (450 k) cohort. **(D)** CD40 and expression in methylation derived clusters from **(C)**. **(E)** Survival analysis of overlapping samples (parametric survival regression, Weibull distribution). **(F)** Cutoff selection of CD40 expression for the separation of clusters. **(G)** Kaplan–Meier survival curve for CD40 expression data with the applied cutoff from **(F)**. **(H)** CIBERSORT estimated immune cell fractions for the RNASeq cohort (upper part, *p*-values: Wilcoxon test) and M2 fractions on CD40 high tumors (lower part). **(I)** Prognostic value of LAPTM5 in CD40 high samples (*p*-value: parametric survival regression).

To validate our hypothesis that *LAPTM5* acts as a potential tumor suppressor in CD40 high-expressing tumors, we evaluated its prognostic value in the TCGA-GBM cohort (Figure 5A). Methylation array data were used to identify similar patients based on associations between methylation patterns and CD40 expression (Figures 5B–D), which were then used to define reasonable cutoffs for CD40 expression based on a ROC analysis (Figure 5F). For the cohort with both methylation and RNASeq data, a survival analysis showed a significant interaction term between the methylation-derived CD40 cluster and *LAPTM5* expression: increasing expression leads to a risk decrease in CD40 tumors in contrast to CD40 low tumors (Figure 5E). Next, the complete RNASeq cohort was evaluated by utilizing the previously derived classification into CD40 low/high tumors. Importantly, neither CD40 nor *LAPTM5* expression alone was able to prognostically separate the patients (Figure 5G, Supplemental Figure 5). In CD40 high tumors, a higher *LAPTM5* expression is associated with better prognosis, while it is associated with a worse prognosis in CD40 low-expressing tumors (Figure 5I, Supplemental Figure 5). To exclude confounding of survival analysis by immune effects, CD40 and *LAPTM5* expression was correlated with the signature of different immune cells. Here higher *LAPTM5* expression was associated with a higher amount of M2-polarized macrophage levels as compared to *LAPTM5* low tumors in CD40 high tumors analyzed by CIBERSORT (32) (Figure 5H).

## Discussion

This study describes the inhibiting effects of *LAPTM5* on tumorigenicity in CD40-positive glioblastoma and its role as a negative regulator of CD40-mediated NFκB signaling. *LAPTM5* relevantly inhibits the clonogenicity and the invasiveness of CD40-positive glioblastoma cells. In addition, as shown *in vitro* and *in vivo, LAPTM5* sensitizes CD40-positive glioblastoma cells to temozolomide treatment, the standard chemotherapy for glioblastoma patients. These *LAPTM5*-induced tumor-suppressing effects are mediated by the inhibition of the CD40-dependent NFκB pathway activation. Finally, a TCGA analysis revealed that a higher expression of *LAPTM5* has a positive effect on survival in CD40-positive glioblastoma patients.

Previous studies in other tumor entities already suggested the potential role of *LAPTM5* in tumorigenesis (8). However, the role of *LAPTM5* had not been assessed in the prototypical invasive tumor glioblastoma so far. We here demonstrate that *LAPTM5* inhibits tumorigenicity in glioblastoma based on functional *in vitro* and *in vivo* experiments and *in silico* analysis. Importantly, we show that the observed *LAPTM5*-mediated tumor-suppressive and temozolomide-sensitizing effects are exclusively observed in CD40-expressing glioblastoma cells, indicating an intact CD40–*LAPTM5* axis as a prerequisite for the tumor-suppressive activity of *LAPTM5*. Silencing of CD40 in CD40-positive U87MG cells abolished the pro-tumorigenic and temozolomide-resistant properties of *LAPTM5* knockdown cells, whereas CD40 overexpression in primarily CD40-negative glioblastoma cells recreated the aggressive phenotype and temozolomide resistance of *LAPTM5* knockdown cells. An interaction between *LAPTM5* and the CD40 receptor was previously shown in B cells (11). Here, for the first time, we could demonstrate the link between CD40 and *LAPTM5* in glioblastoma and characterize the role of the CD40–*LAPTM5* axis in tumorigenicity.

**Figure 6:**
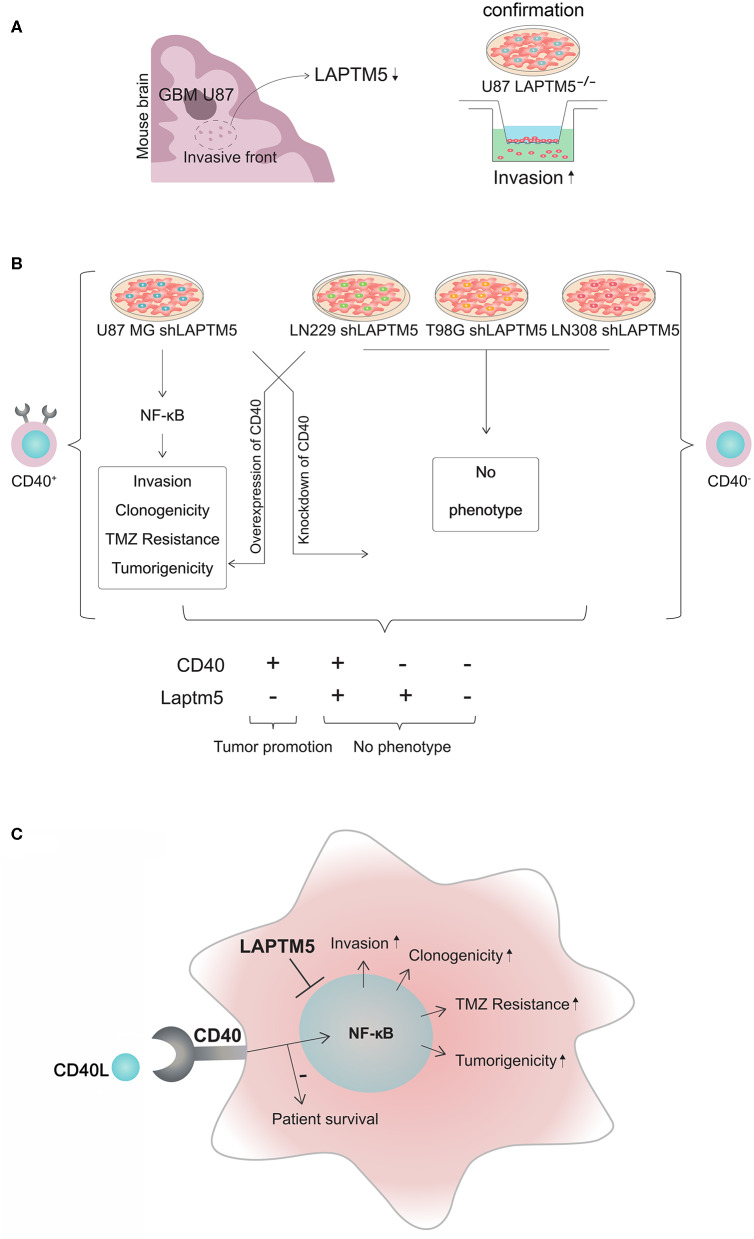
CD40–LAPTM5 crosstalk in glioblastoma invasion and temozolomide resistance. **(A)** *LAPTM5* was found to be downregulated in the invasive front of U87MG cells in an *in vivo* screen for invasion-associated genes. A total of 47,400 transcripts by ~200,000 shRNA sequences were transduced in U87MG cells to identify new invasion-associated genes. *In vitro* experiments confirmed that *LAPTM5*-knockdown U87MG cells were significantly more invasive than the respective vector control cells. **(B)** Overview about CD40-dependent *LAPTM5*-mediated tumor-suppressive effects. *LAPTM5* knockdown resulted in the activation of NFkB pathway in CD40-positive U87MG cells, leading to increased invasion, clonogenicity, tumorigenicity, and temozolomide resistance, which were no longer observed after additional knockdown of CD40 in these cells. Accordingly, the *LAPTM5* knockdown cells had no different phenotype compared to the respective *LAPTM5*-expressing control cells in CD40-negative LN229, T98G, and LN308 cells. Overexpression of CD40 in primarily CD40-negative cells recreated the pro-tumorigenic phenotype and temozolomide resistance of *LAPTM5* knockdown cells. **(C)** Graphic illustration of the CD40–*LAPTM5* crosstalk and its effects in tumorigenicity and temozolomide resistance in glioblastoma. TMZ, temozolomide.

CD40 represents an emerging immune-modulating target in cancer treatment (12). In glioblastoma, CD40-based immunotherapy showed an inhibitory effect on tumor growth in preclinical glioblastoma models *in vivo* (21). Beyond these immune-modulating effects, CD40 signaling was shown to be involved in apoptosis and the neovascularization of glioma cells (37, 38). In this study, we demonstrated that CD40 signaling plays a key role for *LAPTM5*-mediated effects on invasion, clonogenicity, and sensitivity to temozolomide treatment. A further pathway analysis demonstrated that *LAPTM5* mediates these tumor-suppressing and temozolomide-sensitizing effects by the inhibition of CD40-induced NFκB pathway activation. The activation of the NFκB pathway by CD40 signaling has been previously reported in B cells and is consistent with our data (39, 40). Our results indicate that CD40 activates the NFκB pathway in glioblastoma cells only in the absence of *LAPTM5* expression, leading to enhanced tumorigenicity and resistance against temozolomide. As CD40-mediated NFκB activation seems to be required for specific *LAPTM5*-mediated effects, these molecular mechanisms also explain the absence of *LAPTM5*-dependent effects in CD40-negative glioblastoma cells as summarized in Figure 6. In addition, the results are in line with previous studies supporting the role of NFκB pathway activation in promoting glioblastoma cell invasiveness and resistance to alkylating chemotherapies (41, 42).

Personalized therapies based on molecular alterations are increasingly in the focus of neuro-oncological treatment strategies requiring optimal patient selection guided by biomarkers for effective treatment allocation. In this regard, the presented data reasonably suggest *LAPTM5* expression status as a potential biomarker for sensitivity to temozolomide treatment in CD40-positive glioblastoma.

It has been shown that most high-grade gliomas express CD40 with an observed high CD40 expression in about 40% of the tumor samples in patient-derived tumor tissue (37). Although the screening of our glioblastoma cell lines and primary glioblastoma-initiating cell lines revealed only U87MG glioblastoma cells to be positive for CD40 membrane expression, which is in line with previously published data and might be due to a cell culture artifact (37), the expression data of the tumor tissue suggest that CD40 signaling might be relevant for a large subgroup of patients with high-grade glioma. Furthermore, we demonstrated that high *LAPTM5* expression is associated with improved overall survival in CD40 high-expressing glioblastoma. As CD40 is known to be involved in several immune effects, we excluded that the observed association of CD40 high-expressing and *LAPTM5* high-expressing gliomas with better prognosis is related to differences in the signature of immune cells. Here we only found a stronger gene signature for M2-polarized macrophages which are known to be associated with even a worse prognosis (43, 44). Therefore, the positive prognostic value of high CD40 and *LAPTM5* expression is attributed to the tumor tissue and not to the immune cells. Previous studies demonstrated contradicting results about the correlation of CD40 expression with the survival of glioma patients (20, 21). This might be explained by the differences of *LAPTM5* expression in the tumors which were not analyzed in these studies. Of note is that the *in silico* analysis did not reveal a relevant association of CD40 or *LAPTM5* alone with overall survival.

In addition, our data indicate that enhancing CD40 signaling might even have undesirable pro-tumorigenic effects in *LAPTM5*-negative tumors or parts of the tumors such as the invasive front where low *LAPTM5*-expressing cells might cluster due to the pro-invasive phenotype. Here CD40 induction might lead to a further activation of the NFκB pathway and thereby to resistance toward temozolomide treatment and fostering the aggressiveness of gliomas.

In conclusion, we identified *LAPTM5* as a negative regulator of CD40-mediated NFκB signaling in glioblastoma. CD40 activated the NFκB pathway in cells depleted for *LAPTM5* expression, promoting tumor growth and resistance to temozolomide, which could be overcome by NFκB inhibition. The *in silico* analysis revealed that CD40 high-expressing and *LAPMT5* high-expressing gliomas had a better prognosis, which was attributed to the tumor tissue and not to the immune cells. Based on patient survival data and functional *in vitro* and *in vivo* experiments, we concluded that *LAPTM5* acts as a tumor suppressor in CD40-positive gliomas (Figure 6). In addition, we demonstrated an important role of CD40 in *LAPTM5*-mediated effects on tumorigenicity and sensitivity to alkylating chemotherapy beyond the known immune-modulating effects of CD40. Therefore, the data indicated that the focus of CD40-stimulating anti-tumor therapies should not exclusively be on the immune compartment.

As CD40 expression was frequently detected in glioblastoma tissue samples and is a prerequisite for the tumor-suppressive activity of *LAPTM5*, these data provide relevant molecular insights into a potentially new mechanism of resistance against temozolomide in a relevant proportion of glioblastoma patients. Furthermore, based on the presented results, the *LAPTM5* expression status provides a potential new biomarker for response to temozolomide treatment in CD40-positive glioblastoma, which merits further evaluation in clinical practice.

## Data Availability Statement

The datasets generated for this study can be found in the Experiment ArrayExpress accession is E-MTAB-6316.

## Ethics Statement

The animal study was reviewed and approved by Regierungspräsidium Karlsruhe, Karlsruhe, Germany.

## Author Contributions

AB, FB, TK, ZD, and DL provided substantial contributions to the conception, design, acquisition, analysis, and interpretation of data, drafting of the article or revising it critically for important intellectual content, and final approval of the version to be published. ZT provided substantial contributions to the acquisition, analysis, and interpretation of data, and final approval of the version to be published. MK and BW provided substantial contributions to the acquisition of data, analysis of biostatistical data, interpretation of data, and final approval of the version to be published. SP and NH provided substantial contributions to the acquisition, analysis, and interpretation of data and final approval of the version to be published. FW and MP drafted the article or revised it critically for important intellectual content and gave final approval of the version to be published. WW and AA provided substantial contributions to the conception and design, analysis and interpretation of data, drafting of the article or revising it critically for important intellectual content, and final approval of the version to be published.

## Conflict of Interest

The authors declare that the research was conducted in the absence of any commercial or financial relationships that could be construed as a potential conflict of interest.

## Abbreviations

LAPTM5: lysosomal protein transmembrane 5
CD40: cluster of differentiation 40
NFκB: nuclear factor (NF)κB
GIC: glioblastoma-initiating cells
qRT-PCR: quantitative real-time PCR
GSEA: gene set enrichment analysis
LDA: limiting dilution assay
SFI: specific fluorescence intensity
DFS: disease-free survival
TNF-α: tumor necrosis factor alpha.
