# Supplementary material for: LAPTM5–CD40 Crosstalk in Glioblastoma Invasion and Temozolomide Resistance
Source: Front Oncol. 2020 Jun 5;10:747. doi: 10.3389/fonc.2020.00747 (PMC7289993; doi:10.3389/fonc.2020.00747)
Supplement: Supplementary file 1 [file Table_1.DOCX]

**Supplemental Table 1**

**Primer sequences:**

| **Gene** | | **forward (5´- 3´)** | **reverse (5´- 3´)** |
| --- | --- | --- | --- |
| Human LAPTM5 | GTCCCTGCAAATCATGGACT | | AGGTGGGCACTTCCATGTAG |
| Human CD40 | CACAGAGTTCACTGAAACGGAA | | AACCCCTGTAGCAATCTGCTT |
