# Supplementary material for: LAPTM5–CD40 Crosstalk in Glioblastoma Invasion and Temozolomide Resistance
Source: Front Oncol. 2020 Jun 5;10:747. doi: 10.3389/fonc.2020.00747 (PMC7289993; doi:10.3389/fonc.2020.00747)
Supplement: Supplementary file 2 [file Table_2.docx]

**Supplemental Table 2**

**Antibodies**

Primary antibodies

| **Target** | **Source** | **Dilution** | **Manufacturer** |
| --- | --- | --- | --- |
| IkBα | rabbit | 1:1000 | Cell Signaling Technology  Danvers, MA, USA |
| Phospho-IkBα | mouse | 1:1000 | Cell Signaling Technology |
| α-Tubulin | mouse | 1:5000 | Sigma Aldrich |
| LAPTM5 | rabbit | 1:100 | Novus Biologicals  Littleton, CO, USA |

HRP- (horse radish peroxidase) labeled antibodies

| **Target** | **Source** | **Dilution** | **Manufacturer** |
| --- | --- | --- | --- |
| mouse | sheep | 1:5000 | GE Healthcare |
| rabbit | donkey | 1:3000 | GE Healthcare |

Fluophore-labeled antibodies

| **Target** | **Label** | **Dilution** | **Manufacturer** |
| --- | --- | --- | --- |
| CD40 | APC, clone: 5C3 | 1:100 | eBioscience  Thermo Fisher Scientific  Waltham, MA, USA |
| CD40 | FITC, clone: HB14 | 1:100 | BioLegend, San Diego, CA, USA |
