# Supplementary material for: LAPTM5–CD40 Crosstalk in Glioblastoma Invasion and Temozolomide Resistance
Source: Front Oncol. 2020 Jun 5;10:747. doi: 10.3389/fonc.2020.00747 (PMC7289993; doi:10.3389/fonc.2020.00747)
Supplement: Supplementary file 3 [file Table_3.docx]

**Supplemental Table 3**

| No | ILMN Gene | Fold Change U87MG shLAPTM5 vs. RNAi | p.BH | Search Key | Transcript |
| --- | --- | --- | --- | --- | --- |
| 1 | BCL3 | 1,366029713 | 8,4064E-15 | NM_005178.2 | ILMN_17874 |
| 2 | BDKRB1 | 3,602938353 | 6,07523E-39 | NM_000710.2 | ILMN_24608 |
| 3 | CCL2 | 1,742515195 | 1,96521E-08 | NM_002982.3 | ILMN_25185 |
| 4 | CD44 | 1,569089458 | 7,12542E-18 | NM_001001392.1 | ILMN_24089 |
|  | CD44 | 1,333953306 | 1,97054E-22 | NM_001001390.1 | ILMN_10947 |
| 5 | CD74 | 2,293131799 | 2,07641E-27 | NM_004355.2 | ILMN_21746 |
|  | CD74 | 2,208348644 | 8,3194E-31 | NM_001025158.1 | ILMN_21963 |
|  | CD74 | 2,031245231 | 2,64274E-11 | NM_001025159.1 | ILMN_21963 |
| 6 | CSF2 | 3,44289447 | 5,93314E-16 | NM_000758.2 | ILMN_28163 |
| 7 | CSF3 | 12,21045846 | 6,35512E-20 | NM_000759.2 | ILMN_20477 |
|  | CSF3 | 11,74931621 | 1,63321E-22 | NM_000759.2 | ILMN_20477 |
| 8 | F3 | 2,083840054 | 1,29013E-15 | NM_001993.2 | ILMN_182960 |
|  | F3 | 1,717454762 | 1,21474E-08 | NM_001993.2 | ILMN_182960 |
| 9 | IER3 | 2,783679243 | 1,02514E-17 | NM_052815.1 | ILMN_29406 |
| 10 | IL11 | 6,71718567 | 4,74496E-37 | NM_000641.2 | ILMN_23651 |
| 11 | IL15 | 1,874398013 | 1,39814E-10 | NM_172174.1 | ILMN_4978 |
|  | IL15 | 1,696778597 | 3,72293E-10 | NM_172174.1 | ILMN_16803 |
| 12 | IL1A | 2,345891922 | 2,36758E-05 | NM_000575.3 | ILMN_25320 |
| 13 | IL1B | 10,7256176 | 3,49435E-37 | NM_000576.2 | ILMN_27277 |
| 14 | IL6 | 9,555001992 | 1,69158E-26 | NM_000600.1 | ILMN_6469 |
| 15 | IL8 | 3,866355726 | 9,02407E-12 | NM_000584.2 | ILMN_179575 |
|  | IL8 | 3,75847243 | 2,78487E-19 | NM_000584.2 | ILMN_179575 |
| 16 | IRF1 | 2,019320446 | 8,97434E-14 | NM_002198.1 | ILMN_11739 |
| 17 | IRF7 | 1,41340078 | 3,68999E-09 | NM_004030.1 | ILMN_10848 |
| 18 | NFKB1 | 1,416265344 | 8,53235E-16 | NM_003998.2 | ILMN_161884 |
| 19 | NFKB2 | 1,661060256 | 6,79694E-08 | NM_002502.3 | ILMN_26475 |
|  | NFKB2 | 1,578366226 | 1,54938E-14 | NM_002502.2 | ILMN_167932 |
| 20 | NFKBIA | 1,754777424 | 1,11077E-08 | NM_020529.1 | ILMN_6745 |
| 21 | NR4A2 | 2,496808444 | 3,02216E-12 | NM_006186.2 | ILMN_28405 |
|  | NR4A2 | 1,510148722 | 0,000210876 | NM_006186.2 | ILMN_28405 |
| 22 | PTGS2 | 6,29273298 | 9,35195E-09 | NM_000963.1 | ILMN_29986 |
|  | PTGS2 | 5,253314019 | 5,8455E-14 | NM_000963.1 | ILMN_176524 |
| 23 | RELB | 1,731074709 | 2,58135E-14 | NM_006509.2 | ILMN_26715 |
| 24 | SOD2 | 2,291051105 | 1,76228E-07 | NM_000636.2 | ILMN_19880 |
|  | SOD2 | 2,073127661 | 4,21151E-06 | NM_001024466.1 | ILMN_20239 |
|  | SOD2 | 1,85474262 | 2,19239E-06 | NM_001024465.1 | ILMN_19760 |
| 25 | TAP1 | 1,810916563 | 2,28444E-32 | NM_000593.5 | ILMN_17479 |
| 26 | TFPI2 | 2,254529795 | 3,86626E-17 | NM_006528.2 | ILMN_17570 |
| 27 | TGM2 | 2,367170446 | 4,65497E-25 | NM_004613.2 | ILMN_170198 |
|  | TGM2 | 1,298480447 | 4,65144E-09 | NM_198951.1 | ILMN_8134 |
| 28 | TNF | 1,55820445 | 2,14234E-11 | NM_000594.2 | ILMN_24087 |
| 29 | TNFAIP3 | 1,654648669 | 3,61941E-07 | NM_006290.2 | ILMN_2315 |
