# Supplementary figures and images for: LAPTM5–CD40 Crosstalk in Glioblastoma Invasion and Temozolomide Resistance
Source: Front Oncol. 2020 Jun 5;10:747. doi: 10.3389/fonc.2020.00747 (PMC7289993; doi:10.3389/fonc.2020.00747)

Suppl. Fig. 1

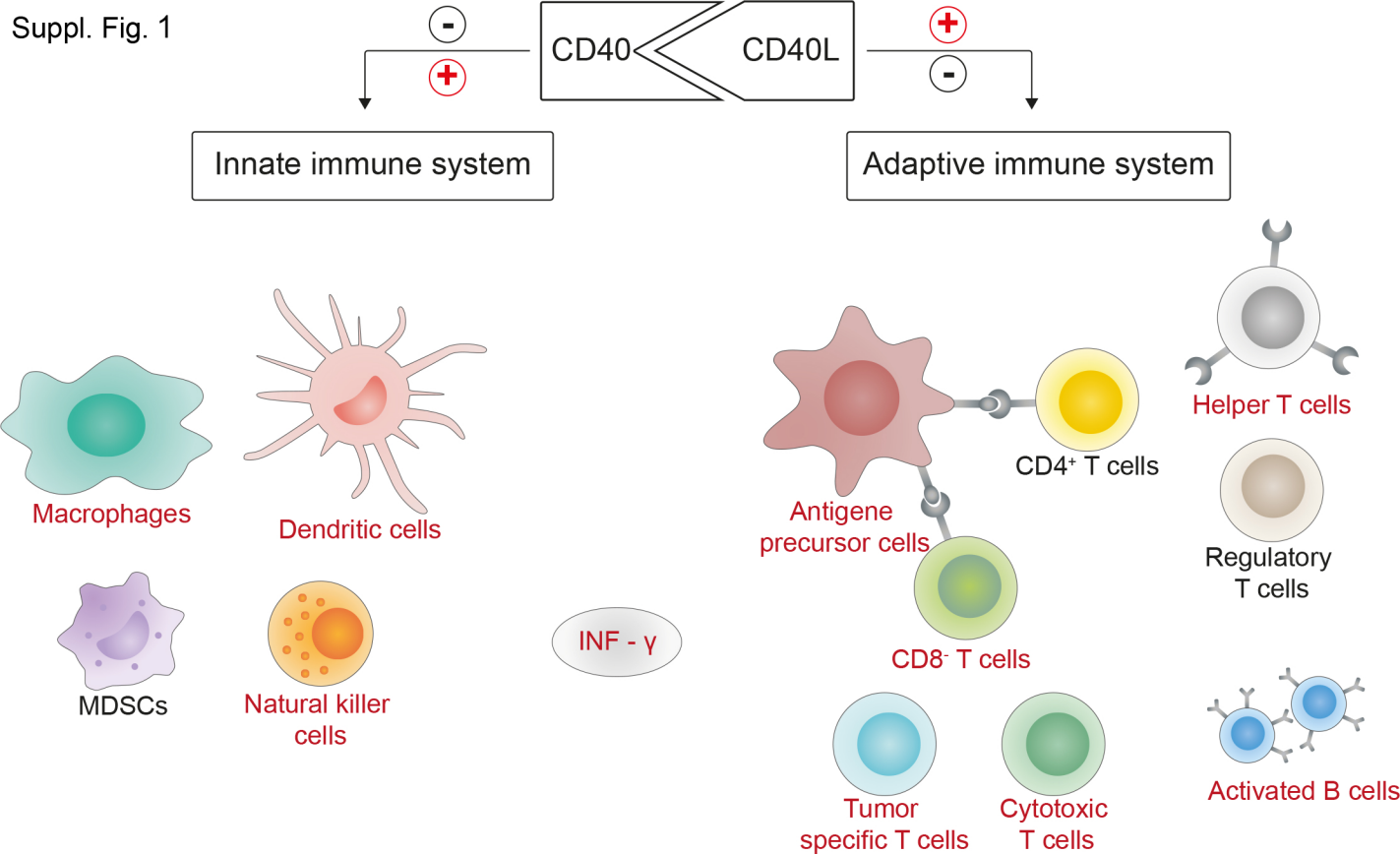

Supplement: Supplementary file 4 [file Image_1.PDF]

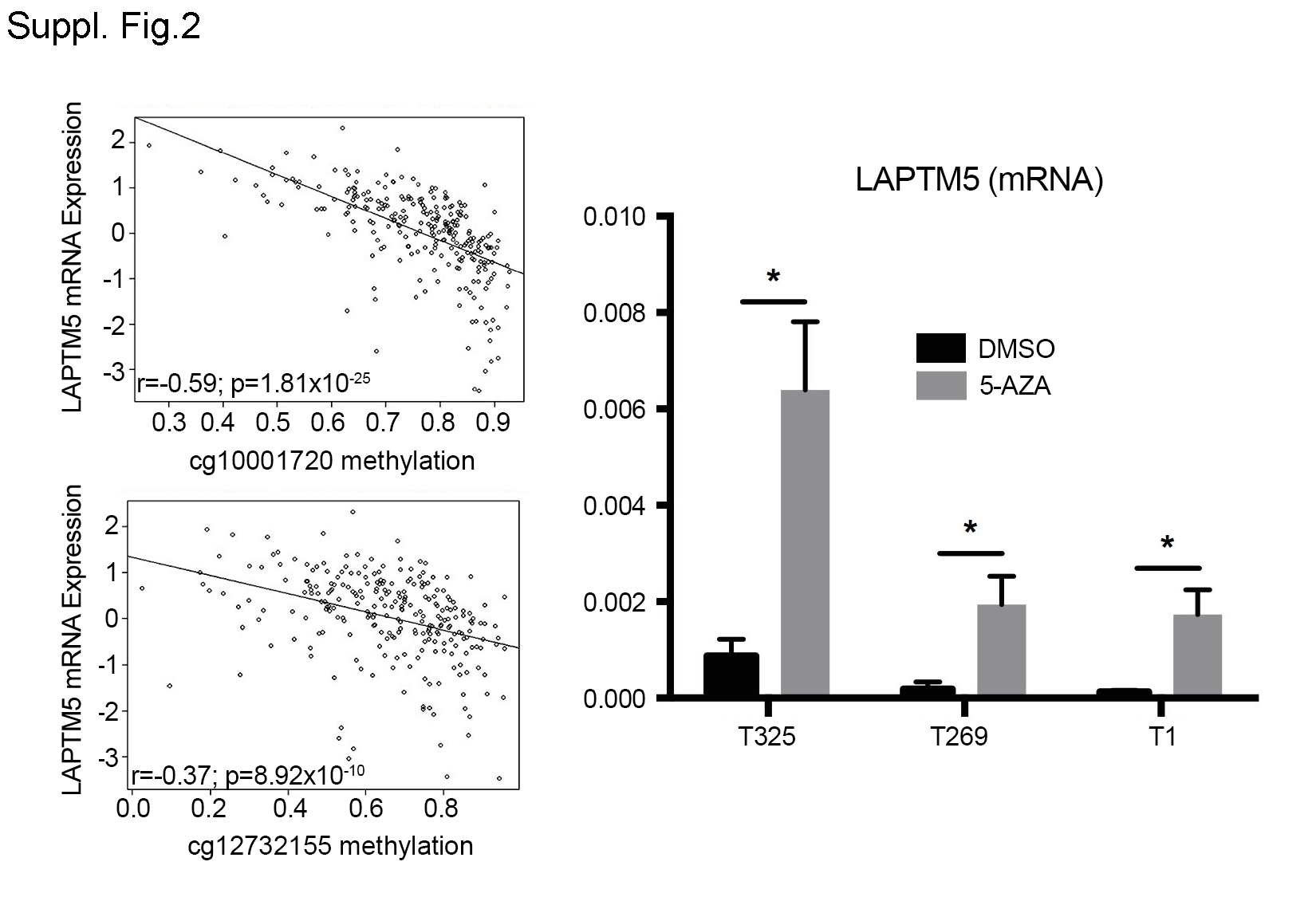

Supplement: Supplementary file 5 [file Image_2.TIF]

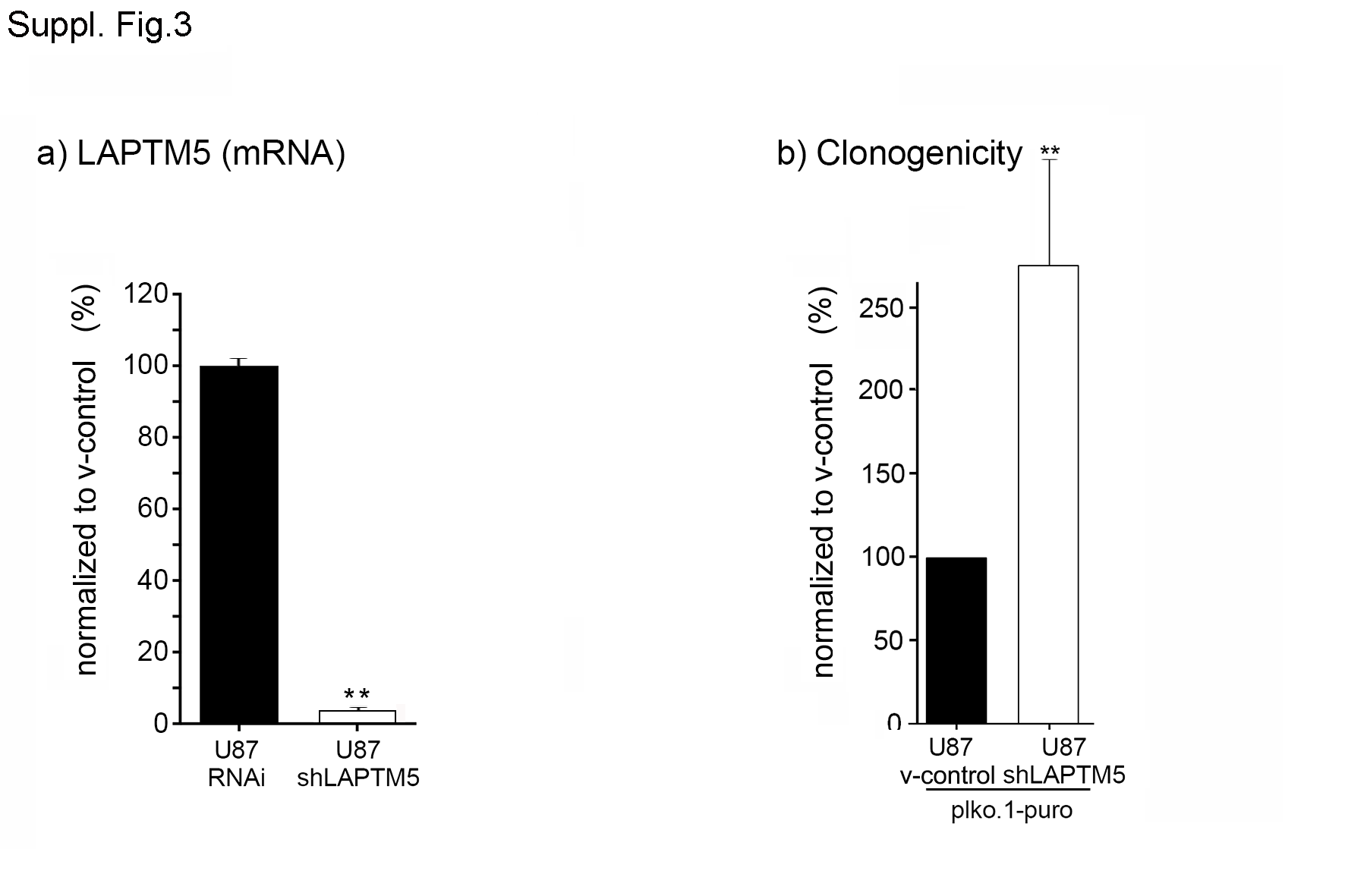

Supplement: Supplementary file 6 [file Image_3.TIF]
