# Supplementary material for: LAPTM5–CD40 Crosstalk in Glioblastoma Invasion and Temozolomide Resistance
Source: Front Oncol. 2020 Jun 5;10:747. doi: 10.3389/fonc.2020.00747 (PMC7289993; doi:10.3389/fonc.2020.00747)
Supplement: Supplementary file 7 [file Data_Sheet_1.docx]

**Supplementary Figures**

**Suppl. Fig. 1:** **Effects of CD40 and CD40 Ligand interaction on immune cells.**

The figure illustrates the known effects of CD40 and CD40L interaction on the innate and adaptive immune cells. Stimulating effects or upregulation of respective immune cells/ cytokines are demonstrated in red color, inhibitory effects in black.

**Suppl. Fig. 2: *LAPTM5* is regulated by methylation.**

**Left:** Correlation of methylation at cg10001720 methylation site (top) with *LAPTM5* expression, pearson’s correlation coefficient (r = - 0.59; p < 0.001). Correlation of methylation at cg12732155 methylation site (bottom) with *LAPTM5* expression, pearson’s correlation coefficient (r = -0.37; p < 0.001).

**Right:** Human glioma initiating cell lines (GICs) T325, T269, and T1 after treatment with 5-Azacytidin (5-AZA) compared to treatment with DMSO control. *LAPTM5* mRNA expression is demonstrated in relation to GAPDH. (* p<0,05).

**Suppl. Fig. 3: Knockdown of *LAPTM5* in U87MG cells**

**a)** Efficacy of *LAPTM5* knockdown was validated by qRT-PCR. *: p<0.05, **: p<0.05. **b)** Functional confirmation of the enhanced clonogenicity in U87 MG *LAPTM5* knockdown cells after use of another *LAPTM5* knockdown virus (plko.1-puro from Sigma Aldrich). Cells show a significantly higher clonogenicity than respective *LAPTM5* expressing (RNAi) cells. Figures represent the mean value of three independent experiments. *: p<0.05, **: p<0.01.

**Suppl. Fig. 4: Validation of CD40 knockdown and overexpression**

**a)** CD40 knockdown was performed in the CD40 expressing U87MG cells and confirmed via qRT-PCR in respective RNAi and *shLAPTM5* cells. **b + c)** CD40 was overexpressed in LN229 **(b)** and T98G cells **(c)**. Overexpression of CD40 was confirmed by increased mRNA expression via qRT-PCR and by increased CD40 membrane staining in flow cytometric analysis (small figures). * p<0.05; ** p<0.01.

**Suppl. Fig. 5: The interplay of CD40 and *LAPTM5* expression influence prognosis in GBM**

Upper row: effect size (survival regression, Weibull distribution) for separation of patients in two groups (high vs low expression) for varying expression cutoffs (positive values: higher risk for high expression). Bottom row: effects as observed for varying cutoffs (left) and Kaplan-Meier survival curves with regression fits for minimal observed p-values (Weibull distribution). Minimum patient group sizes (high / low) are ¼ of the respective cohorts (CD40 high, low, all).

**Suppl. Table 1:**

Primer sequences used for qRT-PCR.

**Suppl. Table 2:**

List of antibodies used for western blot and flow cytometry.

**Suppl. Table 3:**

Single genes upregulated in the TNF-α signaling *via* the NFκB pathway in sh*LAPTM5* cells.
